# The Role of Intra-Tumoral Heterogeneity and Its Clinical Relevance in Epithelial Ovarian Cancer Recurrence and Metastasis

**DOI:** 10.3390/cancers11081083

**Published:** 2019-07-30

**Authors:** Cai M. Roberts, Carlos Cardenas, Roslyn Tedja

**Affiliations:** Obstetrics, Gynecology and Reproductive Sciences Department, Yale School of Medicine, New Haven, CT 06520, USA

**Keywords:** ovarian cancer, intratumoral heterogeneity, clonal evolution, cancer stem cells, tumor microenvironment

## Abstract

Epithelial ovarian cancer is the deadliest gynecologic cancer, due in large part to recurrent tumors. Recurrences tend to have metastasized, mainly in the peritoneal cavity and developed resistance to the first line chemotherapy. Key to the progression and ultimate lethality of ovarian cancer is the existence of extensive intra-tumoral heterogeneity (ITH). In this review, we describe the genetic and epigenetic changes that have been reported to give rise to different cell populations in ovarian cancer. We also describe at length the contributions made to heterogeneity by both linear and parallel models of clonal evolution and the existence of cancer stem cells. We dissect the key biological signals from the tumor microenvironment, both directly from other cell types in the vicinity and soluble or circulating factors. Finally, we discuss the impact of tumor heterogeneity on the choice of therapeutic approaches in the clinic. Variability in ovarian tumors remains a major barrier to effective therapy, but by leveraging future research into tumor heterogeneity, we may be able to overcome this barrier and provide more effective, personalized therapy to patients.

## 1. Introduction

Ovarian cancer remains the most lethal gynecological cancer in United States. It is estimated that there will be 22,530 new cases of ovarian cancer and 13,980 ovarian cancer-related deaths in 2019 in United States [1]. Although platinum-based therapy has been in use for more than four decades, patient survival and prognosis has only been slightly improved [2]. Many studies have asserted that this is because ovarian cancer cannot be defined as a single entity, but rather consists of several different cell types with acquired unique features, conferring heterogeneity to the disease that will dictate chemoresponse and prognosis. 

In ovarian cancer, there are five common subtypes that have been classified based on their histological signatures: high-grade serous carcinoma (HGSC), low-grade serous carcinoma (LGSC), mucinous carcinoma (MC), endometroid carcinoma (EC), and clear cell carcinoma (CCC). Ovarian cancer with epithelial cell origin accounts for more than 90% of all cases and is responsible for the vast majority of the ovarian cancer-related deaths [2]. Therefore, much effort has been invested toward the understanding and treatment of this subtype. In this review, we will focus on ovarian cancer with epithelial origin, particularly HGSC, unless otherwise stated. 

In addition to different subtypes of ovarian cancer, the complexity of the disease is further enhanced by tumor heterogeneity, which can be divided into inter-tumoral and intra-tumoral heterogeneity. Inter-tumoral heterogeneity is defined as those genotypic and phenotypic variations found between multiple tumors of the same type from one patient, e.g., between a primary tumor and a metastatic lesion or between different metastatic lesions. Moreover, the coexistence of different cell populations within one single lesion gives rise to intra-tumoral heterogeneity (ITH). ITH has a crucial role in metastasis, invasion, tumor expansion, recurrence, and therapeutic resistance [3,4]. ITH refers to genomic and biological variations that occur within a single patient, gained by cancer cells, such as cancer stem cells, evolving under specific environmental cues. Heterogeneity between distant lesions can be attributed in many cases to ITH within the site of origin, hence intra-tumoral heterogeneity exists both within and between individual tumor foci. 

ITH arises from clonal expansion driven by genetic modifications through somatic mutations, stochastic genetic changes, or epigenetic alterations. From here, there are two major theories that have been postulated regarding the mechanisms of ITH development, i.e., the clonal evolution and cancer stem cell theories. The behavior of some cancer cells follows the pattern of the stem cell theory, whereby tumorigenic cancer stem cells give rise to a non-tumorigenic (i.e., non-stem) cancer cell population. The clonal evolution model states that populations of tumor cells will acquire unique traits over time as changes accumulate, and spatially and temporally distinct clones result. However, debate persists regarding the contributions of clonal evolution and cancer stem cells to ITH theories, and most likely both mechanisms are at work for a given patient’s tumor(s) [5]. 

While much of the genetic landscape of ovarian cancer has been reviewed extensively elsewhere [6], this review will focus on the sources of ITH in ovarian cancer, evidence in support of clonal evolution and cancer stem cell theories in ovarian cancer metastasis, and the clinical relevance of ITH in ovarian cancer progression. We will dissect key signaling pathways involving cancer stem cell differentiation and highlight how the microenvironment can redefine the course of the disease.

## 2. Sources of Intra-Tumoral Heterogeneity in Ovarian Cancer

### 2.1. Genetic Changes

The genetic changes reported in ovarian cancer include chromosomal instability (CIN) [7,8,9], and some of its manifestations, such as microsatellite instability (MSI) [10]. CIN is a commonly acknowledged hallmark of cancer across many types [11], and comprises changes in ploidy and structural rearrangements of chromosomes [12]. CIN has other manifestations, including the amplification or deletion of key oncogenes or tumor suppressors, respectively, directly contributing to carcinogenesis. Once a cancer is established, continued CIN is subject to selective pressure and can continue to influence the course of disease progression. A recent pair of studies in glioma found that the degree of chromosome 7 copy number variation (CNV) was correlated with tumor grade, likely due to amplification of the epidermal growth factor (*EGFR*) gene [13,14]. Chromosome 7 number varied within the tumor cell population, and this variation was recapitulated in cultures derived from single cells. The authors determined that mis-segregation of chromosome 7 was the reason for this dynamic CIN in the glioma cell population, and daughter cells could change from proliferative to stem-like phenotype based on mitotic errors. High degree of CIN is also implicated in cancer progression; as early as 1976, the aggressiveness and metastatic capacity of melanoma was found to be correlated with CIN [15].

Both aneuploidy and structural rearrangements of chromosomes have been characterized in ovarian cancer, although the abundance of one in general implies lower levels of the other [7]. High grade serous ovarian tumors in particular are marked by high levels of genomic instability [16]. CIN may in fact be an early event in carcinogenesis of ovarian cancer, as one report demonstrated that shear forces from peritoneal fluid may be sufficient to cause chromosome segregation errors, even in non-cancerous cells [17]. It is therefore possible that physical forces from the surrounding environment, in addition to chemical signals, may play a role in tumorigenesis. Furthermore, CIN may arise as ovarian cancer develops due to inherent defects in DNA repair pathways, such as Fanconi anemia [18]. Defects in DNA repair pathways may also play a role in the origin of ovarian cancer. Germline mutations in *BRCA1* and *BRCA2* are known risk factors for ovarian cancer, particularly the HGSC subtype [2]. Other genetic mutations that have been reported to be associated with heredity ovarian cancer syndrome include other BRCA-Fanconi anemia ovarian cancer-associated (*RAD51C*, *RAD51D*, *BRIP1*, and *BARD1*), Lynch Syndrome genes, and mismatch repair genes (*MSH2*, *MLH1*, *MSH6*, *PMS2*, and *EPCAM*), and *STK11* [19].

Due to their role in BRCA1/BRCA2 DNA repair pathway, the loss-of-function mutations of *RAD51C*, *RAD51D, BRIP1*, and *BARD1* genes have been generally considered as ovarian cancer susceptibility genes. Deletion of *RAD51C* and *RAD51D* alleles has been reported to moderately increase the lifetime ovarian cancer risk by 5–15% [20,21], but not *RAD51B* [22]. The deleterious mutation of *BRIP1* (BRCA1-interacting protein C-terminal helicase 1), which aids unwinding DNA for repair, is associated with increased risk of ovarian cancer, in particular HGSC tumor phenotype [19]. *BRIP1* frameshift mutation, c.2040_2041insTT, is estimated to increase the risk of ovarian cancer by 8.1-fold [23]. Another frameshift mutation in *BRIP1*, c.1702_1703del, may also contribute to increased risk of ovarian cancer [23], although a larger sample size is needed. BRCA1-associated RING domain protein 1 (*BARD1*) is known to form heterodimers with BRCA1 and stabilize each other. A germline mutation in *BARD1*, Q564H, has been reported to be associated with ovarian cancer, as well as breast and endometrial cancer [24]. This mutation inhibit its binding to mRNA polyadenylation factor, CstF-50, which resulted in reductions of mRNA 3’ end formation (poly-adenylation) at sites of DNA damage and reduces its tumor suppression functions [25]. On the other hand, genetic mutation of *PALB2* (another BRCA-Fanconi anemia ovarian cancer-associated gene), whose transcribed protein binds at BRCA1 and RBCA2 at the sites of DNA damage, has been associated with heredity of breast cancer syndrome, but not with ovarian cancer. Metcalfe et al. 2017 reported that from 429 women with ovarian cancer, there was no *PALB2* mutation being detected [26]. 

DNA mismatch repair deficiency, whether due to germline mutation or other mechanisms of gene silencing, is known to cause heredity of ovarian cancer syndrome. Although germline mutations in *MSH2*, *MLH1*, *MSH6*, or *PMS2* only account for 2% of hereditary ovarian cancer, the loss of function of these genes by other mechanisms accounts for at least 29% of ovarian cancer cases. *MLH1/MSH2* or *MSH6* mutation carriers have an increase of ovarian cancer risk by 4–24% and 1–11%, respectively [27]. Impaired DNA mismatch repair due to alterations of *MLH1* or *MSH2* increase the chance of mutation in the repetitive DNA sequences (microsatellites) that are located within either coding or non-coding region of the genome [10]. Therefore, it could potentially contribute to the ITH within ovarian cancer not only through the mutations itself, but also endless combinations of MSI resulting from these mutations. MSI, i.e., changes in repetitive elements mostly due to defects in mismatch repair, is commonly associated with colorectal cancer [28]. However, MSI has been reported in several other cancer types including ovarian. In addition, a variant of MSI known as elevated microsatellite alterations at selected tetranucleotides (EMAST) that appears independent of mismatch repair has been reported. EMAST in lung cancer was correlated with p53 mutation [29], a common feature of HGSC tumors. Whether this form of MSI is also relevant to ovarian cancer requires further analysis; although a prior study found only 13% of serous ovarian tumors analyzed contained EMAST, all EMAST+ samples were of advanced stage [10].

Finally, as mentioned above, alterations in the tumor suppressor gene p53 are near ubiquitous in HGSC, appearing in approximately 96% of all patient tumors [30]. However, these mutations are not associated with high risk hereditary ovarian cancer syndrome. In contrast, mutations in the SWItch/Sucrose Non-Fermentable (SWI/SNF) gene *ARID1A* are considered driver mutations in approximately 50% of ovarian clear cell carcinomas and 30% of endometrioid tumors [31]. As SWI/SNF family members are required for chromatin remodeling, mutations in these genes may link genetic changes to epigenetic alterations, discussed further below. 

### 2.2. Epigenetic Changes

In addition to, and sometimes as a result of genetic alterations, epigenetic changes can also contribute to tumorigenesis and ITH. Epigenetic changes include direct methylation of DNA, posttranslational modifications of histones, or posttranscriptional regulation of mRNA, such as by microRNAs (miRNAs) [32]. It is well noted that epigenetic modifications, like DNA methylation, occur in normal embryonic development [33,34]. Methylation in normal embryonic development is tightly regulated and relatively stable, while methylation in cancer is random and unstable. The target site of DNA methylation is a CG rich region, known as CpG. CpG regions are not distributed equally within the genomic sequence; regions with high frequencies of CpG are referred to as CpG islands. There are two main ways in which DNA methylations can facilitate cancer formation: (1) hypermethylation and (2) hypomethylation. Hypermethylation in CpG islands of gene promoters in close proximity to the start codon results in transcriptional repression of these genes. On the other hand, hypomethylation of highly repeated DNA sequences can result in overexpression of nearby genes. A generalized portrait of what genes are likely to be differentially epigenetically regulated in ovarian cancer cell populations has been hindered by the relatively small number of studies and small numbers of samples within those studies. However, there is evidence that changes in DNA methylation may give rise to initiation of ovarian cancer development and therapy-resistant cells, and that targeting epigenetic effectors may reverse this [16]. In particular, inhibitors of histone modification proteins have entered clinical trials.

In epithelial ovarian cancer, random methylation of multiple CpG islands has been reported to occur more frequently when compared to the normal ovarian surface epithelium. Hypermethylation of CpG islands of *OPCML*, *DCR1*, *RASSF1A*, *BRCA1*, and many other genes have been detected in early stage of ovarian cancer [35]. Hypermethylation of the *BRCA1* gene resulted in its down-regulation and therefore impaired DNA repair, which again similar to *BRCA1* mutations, may contribute to chromosomal or genetic instability and ultimately increase ITH in ovarian cancer. Hypermethylation of cell cycle control genes (*PTEN* and *RASSF1A*) has been reported in epithelial ovarian cancer [36,37]. Additionally, epigenetic changes may help ovarian cancer cells to escape cell death pathways in response to chemotherapy, via hypermethylation of pro-apoptotic genes, including *LOT1* [38], *DAPK* [39], *TMS1/ACS* [40] and *PAR4*. Furthermore, hypermethylation of cell adhesion related genes, such as *ICAM-1* [41] and *CDH1* [42], in ovarian cancer could also facilitate cell migration and metastasis. 

In addition to hypermethylation, hypomethylation has been detected more recently in ovarian cancer, which may result in DNA instability by increasing the frequency of mutations and thus cancer risk. In 2009, a large study of 148 healthy individuals and 113 age-matched pre-treatment ovarian cancer patients was performed to measure the level of methylation of over 27,000 CpGs in blood cells using genome-wide DNA methylation profiling [43]. This study identified as much as 2714 cancer related CpG methylation changes, and 56% of these were hypomethylated. Moreover, more than 40 out of 50 CpGs with highest correlation to cancer were hypomethylated in ovarian cancer patients [43]. For example, hypomethylation of Satellite 2 (Sat2) DNA in the juxtacentromeric (centromere-adjacent) region of chromosome 1 has been highly correlated with poor prognosis in ovarian cancer [44]. 

In addition to hyper- and hypo-methylation, other epigenetic mechanisms, including histone modification and miRNAs, have a significant contribution to ITH in ovarian cancer. Importantly, expression of epigenetic factors can have pleiotropic effects across multiple cancer-associated pathways. For instance, there is increased evidence that over-expression of the polycomb group protein enhancer of zeste homologue 2 (*EZH2*) positively correlates with worsening histological grade and advance stages of ovarian cancer [32]. This may impact BRCA1 activity, signaling via transforming growth factor (TGF)-β1, and even the stem cell state of the tumor cells [45,46,47]. Another epigenetic factor, Sirtuin-1 (SIRT1), a histone and non-histone deacetylase, has been reported to be overexpressed more prominently in early stages of ovarian cancer [48], and is associated with poor prognosis [49]. SIRT1 may contribute to ovarian cancer development by regulating DNA repair and metabolism through inactivation of p53. This allows cells with damaged DNA to overcome cell-cycle control, escape apoptosis, and accumulate mutations that contribute to ITH. SIRT1 is also known to have a significant role in induction of epithelial to mesenchymal transition (EMT) and therefore further contribute to ITH in ovarian cancer. Genetic instability, EMT, and cancer stem cells are all important considerations in how ovarian tumors evolve and respond to therapy, as described below, and the ability of even a single epigenetic factor to touch on all three mechanisms speaks to the importance of including epigenetic changes in any description of ovarian cancer ITH.

## 3. Clonal Evolution Theories in Ovarian Cancer

As a result of the above processes, distinct populations of cells can arise in a number of ways, including: (1) differentiation from clonal evolution, (2) the existence of cancer stem cells, and (3) influences of the tumor microenvironment. We will focus here on clonal evolution; discussions of the other mechanisms appear in subsequent sections.

Cancer development has long been believed to rely on genetic instability, as cells accumulate somatic genetic alterations in the process of clonal evolution [30]. Clonal evolution is based on the Darwinian theory, in which cells have to acquire genetic changes to be able to resist anoikis, migrate, and form tumor foci in secondary organs. In cancer metastasis, there are two possible mechanisms: linear and parallel clonal evolution. In linear clonal evolution, the sub-population with metastatic potential arises as the disease progresses to later stages, while parallel clonal evolution suggests that a sub-population of cells acquire the ability to metastasize early on, separating from the primary tumor at an early stage and upon reaching a secondary site, evolve independently of the primary tumor [50,51]. 

Recently, the origin of ovarian cancer has become a major topic of debate. Increasingly, groups have found that what presents as ovarian cancer clinically may in fact consist of cells originating from the fallopian tube epithelium as opposed to the ovarian surface (as reviewed in [52,53]). That tumors such as these exist lends credence to the idea of parallel evolution, as a cell population would have to gain migratory potential quite early in the disease to colonize the ovary as a pseudo-primary tumor. Further studies suggest that heterogeneous metastases may be the result of multiple distinct populations breaking off both physically and genetically from the primary tumor at different time points [51].

In ovarian cancer, the importance of genetic and epigenetic changes to facilitate metastasis is still debated. Metastasis in ovarian cancer has long been believed to occur through exfoliation of tumor cells from the primary tumor, taking advantage of peritoneal fluid movement, followed by invasion within the peritoneal cavity. What is less clear is the identity of these founding cells. Interestingly, work by Khalique et al. demonstrated that distinct metastatic lesions from the same patient showed different genetic profiles, although any given metastasis was less heterogeneous than its corresponding primary tumor [51]. These findings imply that several cells or populations of cells independently gave rise to metastases, supporting a parallel evolution model. Even in 1982 it had been reported that in melanoma, metastatic lesions could arise from distinct clones, and that the various clones present in a primary tumor differed in their metastatic capacity [54]. Furthermore, the presence of some identical genetic profiles between metastases suggests limited clonal evolution following colonization at the secondary site. An in-depth analysis by McPherson et al. demonstrated that when comparing multiple tumor foci in a single patient, most metastatic lesions are the products of a pure population or very closely related populations of cells, suggesting limited mixing of cells or evolution at a given site. However, there are some tumors that show evidence of polyclonal migration of cells and/or mixing of cell populations in the peritoneal cavity [55]. It has been shown that in some cases, metastasis is not a unidirectional process; rather, cells from distant sites can “self-seed” the primary tumor [56]. Indeed, McPherson et al. found that a small subset of ovarian tumors studied displayed this phenomenon, further complicating lineage analysis of disseminated ovarian tumors [55]. It is possible that differences in clonality of metastases may be the result of the timing of metastatic spread. A study using the Confetti mouse lineage tracing system determined that in squamous skin carcinoma, metastases only showed multiple colored cells if labeling was done after carcinomas had started to progress, mix, and invade at the primary site [57]. Application of this mouse model in ovarian cancer model systems may shed new light on the dynamics of metastasis and clonal evolution.

**Figure 1:**
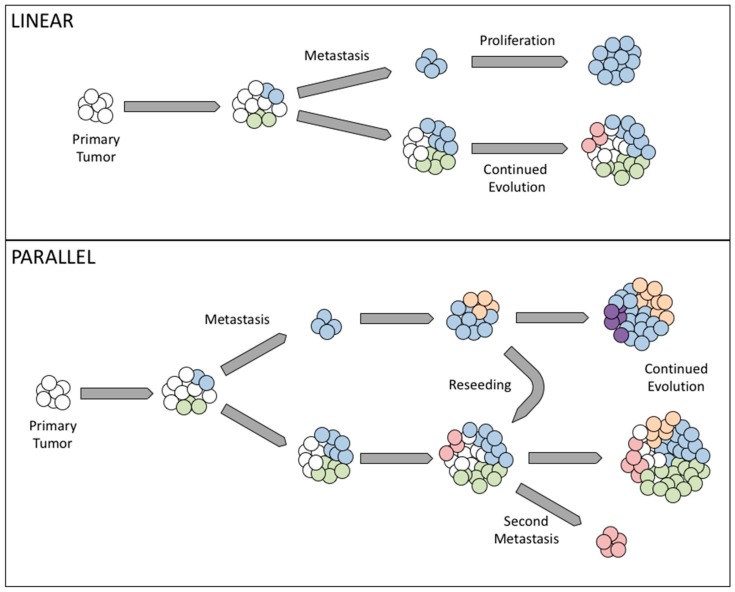
Comparison of linear and parallel forms of clonal evolution. Upper panel: In linear evolution, distinct populations of cells arise in the primary tumor (blue, green). Cells capable of metastasis break off and form monoclonal tumors in secondary sites (top blue clone). The primary tumor continues to evolve new populations (red). Lower panel: In parallel evolution, Multiple populations capable of metastasis arise at different places and times (blue, red). Both primary tumors and metastases continue to evolve, with new populations from metastases (orange, purple) arising and even reseeding the primary site (orange).

In summary, both linear and parallel forms of clonal evolution have been observed in clinical ovarian cancer cases, and indeed both may be present at different sites within a single patient. Linear evolution explains those cases where metastatic tumors appear largely homogeneous, while parallel evolution combines multiple metastatic populations with the ability to seed several sites independently (Figure 1). 

## 4. The Role of Cancer Stem Cells in Ovarian Cancer

### 4.1. Identifying Ovarian Cancer Stem Cells

Apart from the clonal evolution processes, cancer may also develop from cells with stem cell characteristics, alternately termed tumor-initiating cells or cancer stem cells (CSCs). These cells may arise from (1) a stem cell that acquires mutations and over-enhances self-renewal mechanisms or (2) a somatic cell that has acquired genetic changes due to cues from the surrounding environment, gaining stem-like properties, including but not limited to asymmetric division. Virchow first proposed cancer stem cells as the origin of cancer development in the 1850s [58]. He described how cancer could develop via activation of dormant immature cells present in adult tissue. The existence of cancer stem cells was not proved until 1994 in a human acute myeloid leukemia model. The cells with surface markers CD34+/CD38− were reported to be able to closely mimic almost all the features of the disease upon transplantation into mice, while neither CD34+/CD38+ nor CD34– cells had similar behavior [59]. Along with many other subsequent findings, this study further confirms the existence of a specific population of cells that can give rise to all other subpopulations of cells, which significantly contribute to intra-tumoral heterogeneity [60,61,62,63]. Hereafter, we refer to cells that have this ability as CSCs; those than lack this ability, and often make up the bulk of a tumor, are considered non-stem cells.

Ovarian cancer stem cells were first isolated and characterized from ascites fluid from a patient with stage IV ovarian cancer by Bapat and colleagues in 2005 [64]. They were able to propagate more than 10 clones that pass selection processes, based on the cells’ ability to resist anoikis and form spheroids, self-renew (by expression of KI-67), and express stem cell markers (e.g., NESTIN, NANOG, CD44, and OCT4) [64]. There are an ever-growing number of studies following this finding on ovarian cancer stem cells. There are different surface markers used to isolate ovarian cancer stem cells, including CD133+ [65,66,67], CD133+/ALDH+ [68,69], CD44+/MYD88+ [70,71], CD44+/CD117+ [72], CD44+/CD24+ [73], and many others. Additional studies have suggested EpCAM, LGR5, and LY6A as putative markers of CSCs and CD24 as a marker of metastatic cells [74,75]. In addition to different marker profiles, CSCs have been found that exhibit varying morphology. Alvero et al. have identified a population of CSCs that exhibit epithelial morphology and that express epithelial markers [70]. A following discovery by Ho et al. identified two populations of cells present in ascites, one epithelial and one mesenchymal in appearance. Both expressed CSC markers such as CD44, but also expressed genes associated with metastasis, although the authors acknowledge that the mesenchymal-like population may represent tumor associated stroma [76]. Taken together, these studies thus far clearly suggest there exists huge heterogeneity in ovarian cancer stem cells between different studies and perhaps between different patients. 

The heterogeneity within the CSC population could be a result of their different origin and/or the clonal evolution (genetic/epigenetic changes) during tumorigenesis and tumor progression. The origin of ovarian cancer stem cells is still debated. CSCs may originate from normal stem cells within the ovary that acquire genetic/epigenetic changes, or from an ovarian cancer cell that differentiates in response to the tumor microenvironment and acquires stem-like properties. Parte et al. describe separate populations of ALDH1/2+ CSCs that differ in their expression of the proliferation marker KI-67. Interestingly, ALDH1/2 expression was also found in morphologically normal ovarian tissue, suggesting that CSCs may arise from normal stem cells [74]. Kenda Suster et al. also argued for ovarian surface epithelial stem cells giving rise to CSCs, having found a population of morphologically distinct cells within this niche that expressed pluripotency markers such as NANOG, SSEA4, and SOX2 [77]. 

Secondly, ovarian CSC population heterogeneity could also result from clonal evolution in cancer stem cells. One study in clear cell carcinoma revealed that distinct CSC populations from a single patient differed in their ability to give rise to tumors in an in vivo environment, with some requiring a humanized niche for growth while others could grow in a murine niche [78]. WNT, NOTCH, and Hedgehog signaling were shown to be required for the maintenance of proliferative and self-renewal capacities, emphasizing how interactions with the microenvironment can influence stem cell behavior [5].

### 4.2. Role of Cancer Stem Cells in Metastasis and Recurrence in Ovarian Cancer

**Figure 2:**
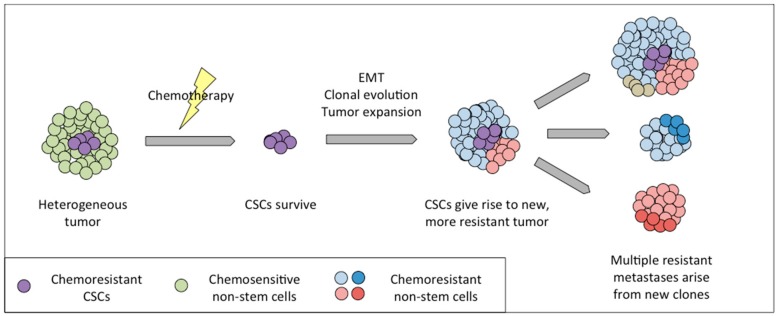
Contribution of cancer stem cells (CSCs) to recurrence and metastasis. A tumor with populations of CSCs (purple) and non-stem bulk tumor cells (green) treated with chemotherapy may eliminate all fast dividing cells, leaving resistant CSCs behind. CSCs will expand and differentiate, undergoing epithelial to mesenchymal transition to give rise to recurrence. Recurrent tumors (red, blue) tend to be resistant to first line chemotherapy, metastasize widely, and continue to evolve (dark red, dark blue), ultimately leading to lethality to the patient.

As we have discussed in the previous section of this review, ovarian cancer metastasis and recurrence are still major clinical problems, as widespread recurrent disease is resistant to the first-line chemotherapy. This chemo-resistant nature may result from residual cancer cells that acquire genetic/epigenetic changes and clonal evolution. Another possibility is that following first-line chemotherapy, the residual disease is only comprised of ovarian CSCs that were resistant to the therapy and give rise to daughter cells with exceptional chemo-resistant characteristics to initiate recurrent disease. During this process, stem cells may undergo genetic and/or epigenetic changes to propagate into new type of cells that differ from the initial bulk tumor, and these cancer cells will display more chemo-resistant characteristics (Figure 2).

Alvero et al. have previously shown that a population of CD44+/MyD88+ cells functions as epithelial CSCs [70]. However, these epithelial cells lack the ability to survive detachment from the primary tumor, instead requiring EMT in order to metastasize. These CSCs are able to undergo EMT in response to over-confluence to give rise to a population referred to as mesenchymal spheroid forming cells (MSFCs) [79]. These cells, which are CD44–, are able to seed metastases in an in vivo xenograft model, lesions which are also entirely CD44–. Therefore, in this model system, the heterogeneity observed between primary and metastatic tumors can be explained by the differentiation of a stem cell population present at the primary site (Figure 2). The CD44– cells that are more susceptible to the first-line chemotherapy are eliminated, while the more chemoresistant CD44+ cells give rise to one or more different cancer cell populations that resist chemotherapy currently available. It should be noted that CIN may result in additional populations of stem-like cells arising that would in turn lead to multiple evolutionary phylogenies, which are not represented in this particular model system.

## 5. Biological Interactions within the Tumor Microenvironment

The tumor microenvironment consists of immune and stromal cells surrounding the tumor, as well as cells that are resident in secondary sites. Cancer cells interact with these cells, exchanging chemical signals and creating a niche where they can survive in new locations. While the impact of non-cancer cell types within a tumor microenvironment to the cancer cells and cancer stem cells is vital to consider, it is also important not to discount the effects of interaction between different sub-populations of cancer cells. Part of ITH is the effect of signaling between subpopulations within a single tumor. However, we will focus here on non-cancer cells. 

In the case of immune cells, CD8+ cells are more abundant in HGSC compared to other subtypes, with increased CD4+ cells and major histocompatibility complex I (MHC-I) expression in recurrent tumors [80,81]. The presence of immune cells will also vary by tumor site within a given patient, and the degree of immune cell infiltration into a tumor and the clonal heterogeneity of that tumor are generally inversely correlated [82]. The reason for this may be two-fold: immune cells may select for one or a few clones at a given site at the expense of immune-susceptible neighbors, or else the presence of many diverse clones may oppose the infiltration of lymphocytes. 

In addition to immune cells, local cell types also interact extensively with tumor cells. In vitro Ingenuity Pathway Analysis studies of two ovarian cancer cell lines revealed that proximity to mesenchymal stem cells upregulated pathways related to cell-cell adhesion and invasion in cancer cells [83]. Similarly, Zhang et al. found that the presence of cancer-associated fibroblasts (CAFs) correlated with metastatic tumor load in patient samples, and likewise encouraged invasion of ovarian cancer cell lines in vitro [84]. The authors also describe CAFs within the metastatic niche in the omentum, with the number of CAFs expanding along with the growth of the tumor. CAFs foster angiogenesis, and may act to prepare a niche prior to arrival of cancer cells, as suggested by the finding that some omental samples contained CAFs but no cancer cells [84]. Thus, it is important to consider that in addition to environments driving changes in tumor clones, the tumor may change the surroundings in return. It is possible that motile clones from the primary tumor create secondary sites with compatible stroma, however another study found that stroma tended to differ between primary and secondary sites, with only platelet-derived growth factor receptor beta (*PDGFBR*) expression in common [85]. Therefore, the balance between tumor evolution to suit a new location and remodeling of target tissues requires further study. 

Another cell type of interest is the adipocytes originating from the visceral fat, as these represent the most common site of ovarian cancer metastasis [86,87] and recurrent disease [88]. Omentum-derived adipocytes have been reported to provide homing for the cancer cells through secretion of adipokines [89]. Moreover, this microenvironment can also enhance tumor growth by transferring FABP4 (a protein that known to regulate lipolysis) to cancer cells, which is used by the cancer cells to process fatty acid and provide cellular energy [89]. Omentum-derived adipocyte stem cells have been shown to promote cancer cells’ resistant characteristic against chemotherapy by modifying the cancer cell metabolism [90]. Our laboratory has previously shown that signaling from the adipocytes via interleukin-6 upregulates *BCL_XL_* in ovarian cancer cells, thus opposing apoptotic signals and driving chemoresistance [86]. These studies clearly indicate that environmental factors at a secondary site have a significant role in inducing distinct clonal evolution and ITH, independent of ITH at the primary site. In addition, ITH in the tumor implant associated with omentum may also be created by genetic/epigenetic changes promoted by adipokine secretion. 

In addition, cancer cells themselves may exert selective pressure on their neighbors. Zhou et al. determined that in glioma, the balance between various cell populations can reach an equilibrium as CIN leads to diverse cell types, but selective pressure from the environment, including paracrine CIN inhibitors, keeps CIN in check [91]. Poste et al. identified a similar phenomenon in melanoma as early as 1981 [92].

Epithelial-to-mesenchymal transition (EMT) is often viewed as a prerequisite for metastatic spread in many solid tumors, including ovarian. This process sees cells downregulate epithelial cell adhesion genes in favor of mesenchymal genes that facilitate motility, invasion, and resistance to anoikis. Local hypoxia or cytokines such as TGFβ can activate signaling cascades culminating in transcriptional reprogramming. Although CIN can contribute to EMT, no genetic changes are necessarily required; alterations in transcription factor expression and activity can be sufficient to effect EMT. For instance, our laboratory has shown that activation of protein kinase C alpha during EMT leads to phosphorylation and stabilization of the protein TWIST1, a master regulator of the EMT transcriptional program [93]. TWIST1+ cells are chemoresistant, and in one model of ovarian cancer, this was shown to be the result of increased survival signaling through the AKT axis [94]. 

Finally, a common feature of ovarian cancer is the formation of ascites. Fluid buildup within the peritoneal cavity provides a unique environment for tumor cells. It has been observed that the tumor cells within this fluid are themselves heterogeneous, and they are exposed to a mixture of pro- and anti-tumor signals from a wide variety of cell types, including cancer-associated fibroblasts (CAFs), endothelial and mesothelial cells, immune cells, and other tumor cells. The composition and balance of soluble factors will change as the disease progresses, but can include interleukins 6 and 8, leading to activation of AKT and survival signaling in floating tumor cells [95]. In addition to free-floating cytokines, tumor cells in ascites are exposed to exosomes from other tumor cells, bearing proteins such as CD24 and the pro-apoptotic proteins Fas ligand and TRAIL [96].

**Figure 3:**
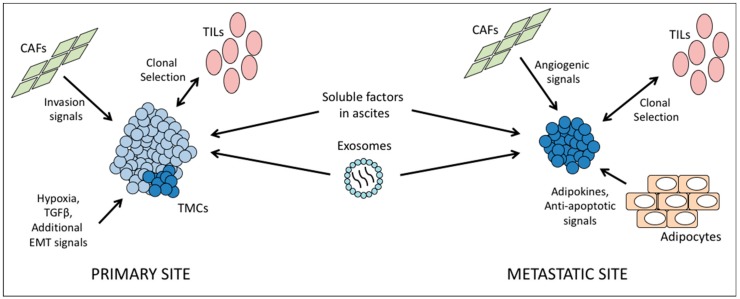
Tumor microenvironment influences tumor cell heterogeneity. Left, at the primary site, hypoxia and epithelial to mesenchymal transition (EMT) signals lead to differentiation of transitional mesenchymal cells (TMCs), a metastatic population. Local fibroblasts regulate adherence and invasiveness of cell populations. Tumor infiltrating lymphocytes (TILs) select clonal populations, while tumor diversity opposes immune action. Center, circulating exosomes and a wide variety of soluble signals from ascites fluid can affect tumors throughout the body and tumor cells in transit. Right, at the metastatic niche such as the omentum, cancer-associated fibroblasts (CAFs) can provide angiogenic signals and prepare a niche for tumor cells. Adipocytes signal to tumor cells and help colonizing cells resist programmed cell death.

Taken together, these data highlight the complexity of microenvironmental factors at each unique tumor site, which must be taken into account in understanding and treating the disease (Figure 3).

## 6. Clinical Relevance of Tumor Heterogeneity 

Tumor heterogeneity provides a wider variety of cells that require elimination for curative therapy. It thus logically follows that ITH is a major barrier to treatment of ovarian cancer. The degree of clonal evolution within and between a patient’s tumors correlates with worse overall and progression-free survival [97], and each topic we have discussed in this review plays a part in this phenomenon.

As mentioned above, the existence of multiple cell populations, each with their own responses to environmental cues and therapy, makes the elimination of ovarian cancer in patients challenging. For instance, standard of care, platinum and taxane drugs, is often effective initially but fails upon recurrence. Cancer stem cells, due to quiescence or intrinsic drug resistance, may survive initial therapy and repopulate the tumor with chemoresistant progeny (Figure 2). In support of this view, Liu et al found that the prevalence of CD44+/CK19+ CSCs was associated with decrease progression-free survival, while Steffensen et al. found that CSCs, as identified by the same markers, led to increased recurrence in early stage tumors [98,99]. Overcoming recurrence will therefore partially depend upon novel therapeutic approaches that are able to reverse chemoresistance and target CSCs. For instance, a recent study in breast cancer determined that a novel agent that activated reactive oxygen species-dependent ferroptosis was more effective against breast cancer stem-like cells [100]. Two additional studies in HGSC found that delivery of small interfering RNA against TWIST1, a transcription factor implicated in chemoresistance and EMT, sensitized ovarian tumors to platinum drugs in an in vivo model [101], and anti-tumor efficacy was further improved if nanoparticle carriers were coated in hyaluronic acid and therefore targeted to CD44+ cells [102]. In addition, more recently the CSC phenotype has been linked with induction of EMT, which results in migratory capacity and metastasis. Therefore, a combination of a CSC-targeting agent and other approaches such as surgery, chemotherapy, immunotherapy or microenvironment-targeted agents maybe a better treatment to eradicate the tumor growth. Encouragingly, targeting of EMT-linked pathways such as WNT, NOTCH, and/or Hedgehog has shown anti-CSC activity in a number of cancers, as reviewed in [103]. 

Whether focused with CSCs or not, it is important to consider that treatment may itself be a selective pressure that drives clonal selection. Populations that were once minor may become major contributors to recurrent tumors following elimination of other phylogenies by therapy, as has been shown in paired pre-and post-therapy clinical samples [97]. Dynamic CIN may also allow for the reestablishment of a new equilibrium between cell populations after therapy has reduced these pools, as seen previously in glioma [91].

ITH also lies at the heart of resistance to second line or targeted therapies. Approximately 15% of epithelial ovarian cancers display mutation or loss of *BRCA1* or *BRCA2*, leading to susceptibility to DNA damaging agents [104]. Such tumors are particularly susceptible to a synthetic lethality approach using inhibitors of Poly (Adenosine diphosphate-ribose) polymerase (PARP), a family of enzymes required for recruitment of DNA repair machinery to sites of damage [105,106]. Therapeutic use of PARP inhibition was as first proposed in 2005, and since then PARP inhibitors (PARPi) have entered clinical use [107,108]. However, resistance is common. The main mechanism is a secondary mutation in BRCA restoring wild type function and rescuing synthetic lethality [109]. As reviewed by Gogola et al., additional genetic or epigenetic changes may yield the same result, such as loss of methylation of the *BRCA* promoter or genome rearrangements leading to *BRCA* overexpression [105]. Additionally, some PARPi may be expelled from target cells due to upregulation of drug efflux pumps [110], which has been detected in some ovarian tumors [111]. Finally, downregulation of *PARP* itself may provide a means of resistance [112]. While a tumor may initially be sensitive to PARPi, clonal evolution may yield populations of cells that have undergone any of these changes, meaning these cells will survive therapy and give rise to refractory recurrence. Interestingly, one study found that multiple different reversion events occurred in a single patient, again highlighting heterogeneity as a challenge to be overcome [111]. Strategies to overcome PARPi resistance are mostly focused on renewing synthetic lethality; drugs inhibiting other DNA repair factors may restore “BRCAness” in tumors [105]. Alternatively, PARPi resistant tumors may be susceptible to ionizing radiation, and thus for tumors where novel reverted clones have been identified, radiotherapy may be indicated. 

In addition to PARPi, the synthetic lethality (SL) approach, as well as the related phenomenon synthetic dosage lethality (SDL), may be used to exploit other defects that arise as a result of CIN in ovarian cancer. SL occurs when perturbation of two genes can induce cell death, while perturbation of one of the genes alone is not affecting the viability of the cells. While SDL arises when down regulation of one gene and overexpression of another gene is become lethal for the cell. *PARP* is but one example among many CIN-related gene targets for which drugs have reached or passed through the clinical trial stage. A large number of studies have identified SL and SDL relationships between genes required for DNA replication and repair, cell cycle progression, and epigenetic remodeling (reviewed in [113]), which may provide ample opportunities for further drug development to target CIN in ovarian tumors. 

Drug resistance and survival is also impacted by the immune system. Generally, the higher the number of tumor infiltrating lymphocytes, the more favorable the clinical outcome [82]. Increased numbers of CD8+ cells correlate with improved overall survival, while MHC-II expression within the tumor predicts better progression free survival [80,81]. Tumor sites without immune infiltrates, which Zhang et al. refer to as “reservoirs of clonal diversity” may be inherently resistant to therapy in addition to lacking an intrinsic anti-tumor immune response. Tumor sites such as these may explain the general lack of success of PD-1/PDL-1 inhibition as a therapeutic approach in ovarian cancer [82]. 

An additional current topic of research is how increased genomic data on CIN can be brought to bear on treatment selection. Further data towards better understanding on this topic will facilitate the development of novel therapies to fight against chemoresistance. One study of ovarian tumors in the Cancer Genome Atlas found that CIN was associated with changes in cell cycle control and DNA damage response genes, and led to worse overall survival [114]. Intriguingly, the same study found that alterations in *BRCA* correlated with increased copy number variations, but were inversely correlated with mutation load. Furthermore, chromosome breaks tended to be found in similar locations across several tumor samples, but unlike in prostate cancer, no unifying binding site motif could be identified [114,115]. In a separate study, convergent evolution of tumors in a single patient was found to lead to similar chromosome rearrangements in clones derived from different phylogenies [55]. This finding suggests that certain chromosomal rearrangements may be favored in ovarian cancer, and it is possible that targeting these changes may yield novel therapeutic approaches.

In addition to informing novel treatments, CIN may be a biomarker for susceptibility to existing therapies. Penner et al. found that exposure to chemotherapy initially decreased CIN, but increased it in the long term, meaning that chromosomal abnormality load may be a marker for chemoresponse [8]. The authors also found that paired sensitive and resistant cell line models showed less difference in CIN than their primary counterparts, suggesting that in vitro models may underestimate the role of CIN in chemoresponse. Further studies will be necessary to generalize these findings with a greater sample size and variety of tumor types. With further understanding on how CIN impacts chemoresponse, CIN-targeted strategies may emerge that can further improve outcome by overcoming chemoresistance. 

The existence of tumor heterogeneity, both ITH and variation between patients, emphasizes the need for personalized approaches to cancer therapy. Identification of druggable targets in populations of tumor cells that are able to resist first line agents would allow clinicians to find out which existing second line therapies are more likely to be successful, and which clinical trials might be of the greatest benefit to a given patient. In breast cancer, a recent study from Appierto et al. describes a technique single-cell profiling of circulating tumor cells (CTCs) that would permit an overview of the cell populations present in a patient’s bloodstream [116]. Given the correlation of CTCs with poor outcomes in ovarian cancer, such a technique could be adapted for screening the blood or ascites fluid of ovarian cancer patients. In fact, a workflow for such an analysis in ovarian cancer has been published and validated using an established cell line and a cohort of patient CTCs [117]. These techniques would allow researchers and clinicians to detect markers of CSCs, cells undergoing EMT, and expression of potential therapeutic targets in order to trace cell populations over the course of treatment and inform future treatment decisions. Similarly, clinicians may be able to predict resistance to certain therapies based on expression of resistance markers such as drug efflux proteins, or on novel mutations that have arisen (e.g., *BRCA* reversion). Furthermore, another group has published a robust method for establishing primary cell cultures following surgery, from either ascites or solid tumor origin. Although they did not observe significant heterogeneity in functional assays between tumors in the patient samples examined, the authors note that the features examined, such as homologous recombination proficiency, may be common to all tumors if a result of an early event during cancer transformation [118]. Once established, primary cultures could be assayed for expression of whatever targets and biomarkers are of interest. Regardless of the methods used to acquire it, in the fight against ITH, information is likely to be the most powerful weapon in the clinical arsenal moving forward.

## 7. Summary and Conclusions

Despite the breadth of research that has been done and is ongoing in the field, ovarian cancer remains a challenging adversary. Late onset of symptoms and widespread peritoneal metastasis lead to most diagnoses being made at advanced stages. Despite often promising initial responses to chemotherapy, disease relapse is common and often chemoresistant, and therefore fatal. In recent years, targeted therapies such as PARP inhibitors have improved outlook somewhat, but resistance is frequently a problem for these modalities as well. 

The presence of cancer stem cells and multiple phylogenies of tumor cells within a single patient go a long way toward explaining the patterns of recurrence that are seen clinically. While therapies may be effective in eliminating fast dividing tumor cells, cancer stem cells with different characteristics and/or slower growth rates can survive and give rise to recurrent tumors, which may be genetically different from their forebears. The existence of multiple tumor clones may allow multiple rounds of metastatic seeding, creating secondary tumors with distinct traits in distinct sites. Furthermore, even targeted therapies or immunotherapies may only be effective against a subset of tumors or tumor cells, due to novel mutations, epigenetic changes, or effects of local microenvironment on tumor drug response.

Barring a revolution in early detection of ovarian cancers, the key to increased patient survival in the coming years will be dependent upon greater understanding of the heterogeneity within patients’ tumors. Elucidating common genetic and epigenetic changes will allow for the development of new targeted therapies, and analysis of multiple tumor foci in a given patient may allow for more effective combination therapies to be used. Novel biomarkers may allow clinicians to better predict recurrence and metastasis. 

In summary, it is incumbent upon all in the ovarian cancer field to take intra-tumoral heterogeneity into account and leverage our knowledge of this phenomenon into more effective strategies to combat this disease.
